# The emergence of psychotic experiences in the early adolescence of 22q11.2 Deletion Syndrome

**DOI:** 10.1016/j.jpsychires.2018.11.002

**Published:** 2019-02

**Authors:** Samuel J.R.A. Chawner, Maria Niarchou, Joanne L. Doherty, Hayley Moss, Michael J. Owen, Marianne B.M. van den Bree

**Affiliations:** Medical Research Council Centre for Neuropsychiatric Genetics and Genomics, Division of Psychological Medicine and Clinical Neurosciences, Cardiff University, Cardiff, Wales, United Kingdom

**Keywords:** Schizophrenia, Genetics, Neurocognition, 22q11.2 Deletion Syndrome, 22q11.2DS

## Abstract

Individuals with 22q11.2 Deletion Syndrome (22q11.2DS) are at substantial increased risk of psychosis spectrum outcomes including schizophrenia. We conducted a prospective, longitudinal study of the psychopathological and neurocognitive correlates of early psychotic phenomena in young people with 22q11.2DS (n = 75, mean age time 1 (T1) 9.9 years, time 2 (T2) 12.5 years). We also assessed unaffected control siblings (n = 33, mean age T1 10.6 years, T2 13.4 years). The prevalence of psychotic experiences, defined as subthreshold psychotic phenomena, substantially increased in children with 22q11.2DS from 4% (n = 3) in childhood (T1) to 21% (n = 16) in early adolescence (T2) (p = 0.001), and at T2 prevalence was significantly elevated (p = 0.020) relative to control siblings (3%). The emergence of psychotic experiences was associated with levels of childhood anxiety symptoms at T1 and differential development of the attention-executive domain. IQ ability and IQ change, however, were not associated with the emergence of psychotic experiences, indicating that initial changes in attention-executive functioning may precede the decline in global cognition that has been reported to be associated with later stages of psychosis development. Our study highlights that psychotic phenomena emerge early in 22q11.2DS and we implicate attention-executive functioning and anxiety as key domains associated with the development of these psychotic experiences.

## 1 Introduction

Identifying the early antecedents of psychotic disorders is vital for understanding aetiology and informing potential interventions, but does however provide a challenge for researchers. Studies of individuals at clinical high-risk of psychosis, although insightful, capture the end phases of the disease process and are unable to assess earlier premorbid phases. Large cohort studies of the general population have provided insights into the childhood antecedents of psychosis. Children who develop schizophrenia in adulthood have deficits from a young age in verbal reasoning and as they get older they increasingly lag behind their peers in working memory, attention, and processing speed (Reichenberg et al., 2010). However, given the prevalence of psychosis in the general population (2.3–3.5%) (Perälä et al., 2007; van Os and Kapur, 2009), the number of individuals who develop psychosis in cohort studies is small. Studies of high-risk groups, where a high proportion of young people will develop psychotic spectrum outcomes, represents an effective methodology, as individuals can be tracked through the period of increasing psychosis risk.

22q11.2 Deletion Syndrome (22q11.2DS) is one of the strongest risk factors for the development of psychosis, with 30–40% of adults developing psychotic disorder (Monks et al., 2014; Murphy et al., 1999; Schneider et al., 2014). 22q11.2DS affects 1 in 4000 live births and the associated phenotype is highly variable including congenital abnormalities, developmental delay and high rates of psychiatric morbidity across the lifespan (Hiroi et al., 2013; Niarchou et al., 2014; Schneider et al., 2014). Furthermore, the psychosis spectrum phenotype is comparable to that of individuals without the deletion (Bassett et al., 2003; Monks et al., 2014; Tang et al., 2017). Prospective assessment of individuals with 22q11.2DS therefore offers a powerful approach for exploring the early antecedents of psychosis (Gur et al., 2017), particularly earlier stages of psychosis development before individuals meet clinical high-risk criteria and assessment is unbiased by retrospective recall, antipsychotic medication use or the effects of psychosis on the brain. Understanding of these earlier stages will be important for developing very early interventions for psychosis.

Several important insights have arisen from prospective longitudinal studies of children with 22q11.2DS. First, as found in general population studies (Van Os et al., 2000), psychotic features are present on a continuum in individuals with 22q11.2DS (Baker and Skuse, 2005; Weisman et al., 2017) and are predictive of development of later psychotic disorder (Gothelf et al., 2007a; Schneider et al., 2016). Second, longitudinal studies of individuals with 22q11.2DS have found several domains of psychopathology to be associated with psychosis development, including childhood anxiety (Gothelf, Feinstein, 2007a; Schonherz et al., 2014). Third, cognitive development has been associated with psychosis risk in 22q11.2DS, with verbal IQ (VIQ) in particular being implicated (Gothelf et al., 2007b, 2013; Vorstman et al., 2015). Although an under-researched area, the importance of broadly assessing cognition has been recognised. Cognitive flexibility, emotion recognition and reading decoding have been identified as predictors of psychosis development in 22q11.2DS (Antshel et al., 2016), and the authors hypothesised that these neurocognitive changes may be primary and contribute to VIQ decline reported in 22q11.2DS. Fourth, it has been hypothesised that individuals who exhibit cognitive deterioration are particularly at risk of psychosis (Swillen and McDonald-McGinn, 2015). Cognitive deterioration has been reported in children in 22q11.2DS (Duijff et al., 2012, 2013), but may not be specific to the syndrome (Chawner et al., 2017).

Longitudinal studies of 22q11.2DS investigating neurocognitive predictors of psychosis spectrum outcomes to date have focused on late adolescence and early adulthood (see (Tang and Gur, 2017) for a comprehensive review of studies). These periods are very informative for understanding the latter stages of psychosis development and factors predictive of transitioning from high risk-states to psychosis, however, a gap remains in the literature covering earlier periods. There is evidence that early adolescence represents a crucial period, with a large international study of 22q11.2DS reporting that VIQ trajectories of those who later develop psychosis began to diverge from age 11 (Vorstman et al., 2015). Only one longitudinal study has examined neurocognitive and psychopathology domains across this period, assessing children with 22q11.2DS at a mean age of 10 years and then at 13.5 years, and found that baseline IQ predicted later psychotic outcomes (Hooper et al., 2013). However, the authors acknowledge that their sample size was small (n = 42) and that this important period warrants further study. Identifying factors associated with the emergence of very early psychotic phenomena could potentially uncover the initial processes important in psychosis development, without risk of confounding by disease progression. Although early phenomena such as psychotic experiences are transient across adolescence (Niarchou et al., 2015), they do have good short term test-retest reliability (Ronald et al., 2013) and are predictive of adult psychotic disorder in the general population (5- to 16-fold increased risk) (Hanssen et al., 2005; Poulton et al., 2000). It has been argued that schizophrenia and psychotic disorders should be viewed as cognitive illnesses (Kahn and Keefe, 2013), whereby cognitive decline emerges years before the onset of the first psychotic episode (Fuller et al., 2002) and that characterisation of this initial cognitive change is important for diagnosis and understanding aetiology. Examining the neurocognitive factors associated with the emergence of early psychotic phenomena in 22q11.2DS is a unique opportunity to explore this.

## 2 Aims of the study

This study presents findings from the Cardiff University ECHO (Experiences of CHildren with cOpy number variants) study longitudinal cohort of children with 22q11.2DS and their unaffected sibling controls. We have previously reported on cognitive development in this cohort (Chawner et al., 2017), here we focus on the emergence of early psychosis-spectrum outcomes. Our hypotheses were 1) the prevalence of subthreshold psychotic phenomena would increase across early adolescence in children with 22q11.2DS and 2) that the level of impairment and developmental change in specific neurocognitive and psychiatric domains would be associated with the emergence of subthreshold psychotic phenomena in children with 22q11.2DS. In particular, childhood anxiety and development of IQ and executive functions have been previously implicated in the development of psychosis-spectrum outcomes in 22q11.2DS (Tang and Gur, 2017) and the general population (Hall, 2017; Mollon et al., 2018). In light of the limitations of previous studies, we utilised the same measures in cases and controls at both time points, examined cognitive domains beyond IQ, and we also investigated whether individuals who exhibited cognitive deterioration were at increased risk of developing psychotic phenomena.

## 3 Methodology

### 3.1 Participants

This investigation was based on the ongoing Cardiff longitudinal ECHO study. 75 children with 22q11.2DS (mean age in years (SD), time 1 (T1) = 9.9 (2.4); time 2 (T2) = 12.5(2.3), 44 (59%) male) and 33 sibling controls closest in age to the index child (mean (SD), T1 = 10.6 (2.0), T2 = 13.4(2.0), 17 (52%) male) were assessed and thus the study captures the developmental stage of early adolescence. The gap between T1 and T2 is 2.7 (SD = 0.5) years. T1 and T2 age did not differ between 22q11.2DS and controls (independent *t*-test; T1 p = 0.150, T2 p = 0.060). At T1 participants with the deletion were recruited through National Health Service (NHS) medical genetics clinics in the UK, British 22q11.2DS charities Max Appeal and the 22Crew, and rare chromosomal disorder support group Unique. Informed consent was gained from primary carers and participants. Protocols were approved by the NHS Wales Research Ethics Committee. Presence of the 22q11.2 deletion was confirmed via medical genetics clinics and the Division of Psychological Medicine and Clinical Neurosciences laboratory.

### 3.2 Longitudinal assessment

#### 3.2.1 Neurocognitive assessment

To allow comparison of cognitive performance across domains and between individuals, age-adjusted z-scores were derived using norms available for each measure (CANTAB, 2006; Heaton et al., 1993; Wechsler, 1999). Scores were constructed so that a negative score denoted a poorer outcome.

IQ was assessed using the Wechsler Abbreviated Scale of Intelligence (WASI) (Wechsler, 1999), from which Full Scale IQ (FSIQ), Verbal IQ (VIQ) and Performance IQ (PIQ) were derived. These scores are already age-adjusted, subtest age-adjusted scores were derived as well.

Specific neurocognitive domains were assessed using the Wisconsin Card Sorting Test (WCST) (Heaton et al., 1993) where number of perseverative errors measured the set-shifting ability aspect of executive function. Furthermore the following CANTAB (Cambridge Neuropsychological Test Automated Battery) (CANTAB, 2006) tests were administered: spatial working memory (SWM) an executive function task; stockings of Cambridge (SOC) which measured spatial planning and is an executive function task; five choice reaction time (RTI) which measured processing speed; match-to-sample task (MTS) a test of visual attention; and rapid visual information processing (RVP) a measure of sustained attention. For MTS, only raw scores were used because there are no CANTAB norms for this task.

### 3.3 Psychiatric assessment

#### 3.3.1 Psychosis assessment

We assessed psychosis-spectrum-outcomes using the psychosis section of the Child and Adolescent Psychiatric Assessment (CAPA) (Angold et al., 1995) that probes for the presence of psychotic disorders and broader subthreshold phenomena, which we have termed “psychotic experiences”. This measure is especially designed to be a child friendly measure of subthreshold psychotic phenomena and has been successfully administered by a previous study of children with 22q11.2DS (Baker and Skuse, 2005). Both primary carer and child report interviews were conducted. In this section, initial screening questions probed for any evidence of perceptual disorders or hallucinations, delusions, or psychotic abnormalities of thought processes. If participants scored on the screening questions, the interviewer continued with more detailed probing about the nature of possible experiences. Questions were asked about the content and location of auditory, olfactory and tactile hallucinations, thought insertion and broadcast, thought echo and withdrawal and delusional thinking. Phenomena were coded in a binary fashion (present/not present), this follows the same approach as a previous study (Baker and Skuse, 2005).

Stringent criteria were applied: a) psychotic experiences were only coded as present if a detailed account of a specific example could be recalled; b) the experience had occurred in the last three months; c) the participant could describe how regularly the experience occurred; d) it was clear to the interviewer that there weren't alternative explanations, phenomena not coded as psychotic experiences included hypnagogic/pompic hallucinations, eidetic imagery, elaborated fantasies, phenomena consistent with the cultural and religious beliefs of the individual's family and peer group, imaginary companions, illusions, hallucinations occurring as part of a seizure or clouded sensorium, spots/stripes before the eyes and sensory changes associated with headaches; e) there had to be agreement by two trained psychologists on the final coding of the audio recorded interview, complex cases were discussed with a consultant child and adolescent psychiatrist. DSM criteria of psychotic disorders were applied to determine whether psychotic phenomena met disorder criteria. In analysis, psychotic experiences were counted as present if reported by either the primary carer or child.

### 3.4 Assessment of childhood psychiatric disorder

Autism Spectrum Disorder (ASD) traits were screened using the Social Communication Questionnaire (SCQ) (Rutter et al., 2003), which was completed by the primary carer. Total scores can range from 0 to 39. In our analysis we used the recommended (Berument et al., 1999) score of 15 or higher as indicating putative ASD.

Psychiatric symptomatology (non-ASD), during the last 3 months was assessed at both times using the CAPA by means of semi-structured interview with the primary carer (parent CAPA). Children aged 11 + completed a self-report semi-structured interview on anxiety and mood (child CAPA). Criteria were applied to establish DSM-IV-TR (American Psychiatric Association, 2000) diagnosis and symptom counts. All interviews were audio recorded and monitored by a consultant child and adolescent psychiatrist. Diagnoses were reached by consensus between two trained psychologists, in cases of disagreement the case was discussed with a consultant child and adolescent psychiatrist.

Individuals were classified as having any psychiatric disorder if they met criteria on either the SCQ or CAPA. For any psychiatric disorder symptom count we added the total CAPA symptom count to the SCQ total score.

## 4 Statistical analysis

### 4.1 Aim 1: longitudinal development of psychiatric disorder and psychotic experiences

For both individuals with 22q11.2DS and sibling controls prevalence and 95% confidence intervals were calculated for each diagnosis. Within each group McNemar's test was conducted to investigate whether prevalence of psychiatric diagnosis and psychotic experiences changed across the two time points. At each time point, Fisher's exact test was conducted to investigate whether prevalence differed between individuals with 22q11.2DS and unaffected sibling controls.

### 4.2 Aim 2: identify factors associated with the emergence of psychotic experiences

Within children with 22q11.2DS we examined whether the presence of psychotic experiences at T2 was associated with the following demographics: age using an independent *t*-test; gender, 22q11.2DS origin (de novo or inherited) and ethnicity using Fisher's exact test; household income and maternal education using Mann-Whitney U tests.

Three children with 22q11.2DS who had psychotic experiences at T1 were excluded from the following analysis to focus on neurocognitive and psychiatric factors associated specifically with the *emergence* of psychotic experiences.

#### 4.2.1 Neurocognitive factors associated with the emergence of psychotic experiences in 22q11.2DS

We investigated whether the presence of psychotic experiences at T2 was associated with cognitive deficits at T1 and T2. Cognitive difference was quantified by subtracting z-scores based on age standardised cognitive performance between those who had psychotic experiences at T2 but not T1 (PE(+)) and those who did not have psychotic experiences at T1 and T2 (PE(-)). To examine whether average cognitive ability and/or change in cognition were related to the emergence of psychotic experiences we first used a Principal Components Analysis approach on 22q11.2DS test-retest age-adjusted cognitive scores, an approach used previously identifying predictors of idiopathic psychotic experiences (Niarchou et al., 2013). This identified two factors s_average_ and s_change_ where s_average_ represents the average cognitive performance across both time points (childhood and early adolescence) and s_change_ cognitive change between childhood and adolescence. This method allows for the examination of change without being influenced by mean performance at either T1 or T2 (Niarchou et al., 2013) and avoiding the issue of mathematical coupling. Logistic regression analysis was conducted for each cognitive measure, with s_average_, s_change_ components as predictors and the presence of psychotic experiences as the outcome.

We also examined whether cognitive deterioration was linked to the emergence of psychotic experiences. Following previous literature, we defined cognitive deterioration as a decrease in raw score between T1 and T2 (Duijff et al., 2012). We then investigated, using logistic regression analysis for each measure, whether cognitive deterioration was associated with the emergence of psychotic experiences at T2.

#### 4.2.2 Psychiatric factors associated with the emergence of psychotic experiences in 22q11.2DS

To test if T1 and T2 psychiatric diagnosis was associated with the emergence of psychotic experiences at T2, Fisher's exact test was conducted. We investigated if symptom count at T1 or T2 differed in those who developed psychotic experiences at T2 using Mann Whitney U-tests. The Principal Components Analysis framework for cognitive scores was applied to psychiatric symptom counts to test for associations between s_average_, s_change_ components and psychotic experiences. Analysis was restricted to those psychiatric features previously found to be significantly more prevalent in children with 22q11.2DS relative to control siblings in the ECHO cohort (Niarchou et al., 2014): any psychiatric disorder (measured by CAPA and SCQ, excluding psychotic disorders and experiences), any non-ASD disorder (measured by CAPA, excluding psychotic disorders and experiences), anxiety disorder, ADHD, ODD, and putative ASD (measured by SCQ).

### 4.3 Statistical correction

Significance thresholds were derived by applying a Bonferroni correction for the number of principal components with eigenvalues>1 derived from T1 and T2 variables. This PCA based method takes into account that variables are not independent of each other due to repeated measures, correlation between cognitive and psychiatric scores, and mathematically related variables i.e. composite scores such as IQ and total symptom count are mathematically related to the subdomain scores that comprise them. For aim 1 the significance threshold was set at p ≤ 0.005; all results greater than 0.005 and less than 0.05 were considered nominally significant. For aim 2 the significance threshold was set at p ≤ 0.0045; all results greater than 0.0045 and less than 0.05 were considered nominally significant.

## 5 Results

### 5.1 Aim 1: psychiatric features across early adolescence

**Fig. 1:**
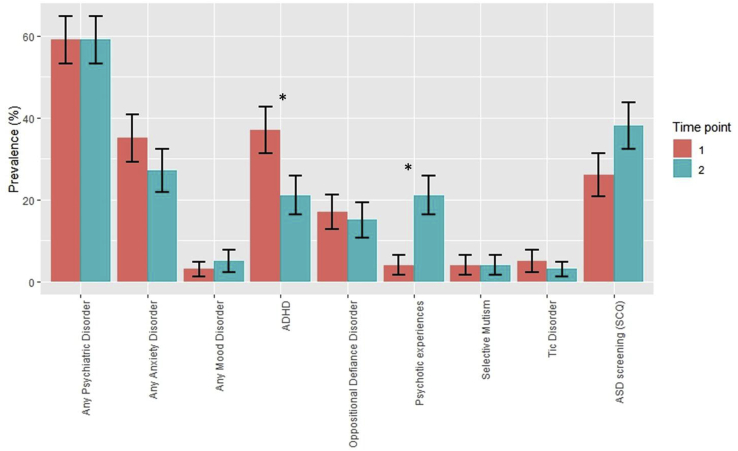
Psychiatric disorders in children with 22q11.2DS at T1 and T2 ADHD, Attention Deficit Hyperactivity Disorder; ASD, Autism Spectrum Disorder; SCQ, Social Communication Questionnaire. See Table 1 for full scores. *indicates significant difference in prevalence (McNemar's p-value <0.05). Error bars represent the standard error.

There was significant psychiatric morbidity in individuals with 22q11.2DS relative to control siblings (see Table 1, Fig. 1). At both time points prevalence of any psychiatric disorder, any non-ASD diagnosis, any anxiety disorder, ADHD and putative ASD diagnosis were elevated in 22q11.2DS. Regarding specific anxiety disorders, children with 22q112.DS had higher rates of generalised anxiety disorder at T2 and of specific and social phobia at T1, compared to control siblings. None of the children (22q11.2DS or controls) met criteria for schizophrenia or other psychotic disorders at T1 or T2. However, the prevalence of psychotic experiences increased in 22q11.2DS from 4% (n = 3) at T1 to 21% (n = 16) at T2 (McNemar's p = 0.001). Prevalence of psychotic experiences was elevated at T2 in children with 22q11.2DS relative to control siblings (21% vs 3%, Fisher's p = 0.020), but not at T1 (4% vs 3%, Fisher's p = 0.999). At T2 children with 22q11.2DS reported a broad range of phenomena, including hallucinations across a range of sensory modalities; broader perceptual phenomena such as derealisation, depersonalisation, jamais vu and altered perception of time; as well as delusions and abnormalities of thought processes. The majority of experiences reported at T2 were by the children (15 out of 16 cases), in only 2 out of 16 cases did caregivers report psychotic experiences in their children. The increase in psychotic experiences in children with 22q11.2DS from T1 to T2 was particularly driven by an increase in hallucinations from 2.7% (n = 2) to 16.0% (n = 12) (McNemar's p = 0.010), specifically tactile hallucinations 0.0% (n = 0) to 8.0% (n = 6) (McNemar's p = 0.041) (Supplementary Table 1).

**Table 1:** Psychiatric disorder in 22q11.2DS and unaffected sibling controls at T1 and T2. **Bold** indicates p < 0.05 Any Psychiatric Disorder includes ASD screening. Values for columns 7 and 13 are McNemar p-values and indicate whether there was a significant change in prevalence between time points. Values for columns 14 and 15 represent Fisher's Exact p-values and indicate whether prevalence in children with 22q11.2DS significantly differed from controls.

	22q11.2DS						Controls						Prevalence difference 22q11.2DS vs Controls	
	n	T1%(n)	95%CI	T2%(n)	95%CI	p	n	T1%(n)	95%CI	T2%(n)	95%CI	p	T1 p	T2 p
Any Psychiatric Disorder	69	59% (41)	48,71	59% (41)	48,71	0.999	30	7% (2)	−3,2	7% (2)	−3,2	0.999	<0.001	<0.001
Any Non-ASD Psychiatric Disorder	75	53% (40)	42, 65	48% (36)	36, 60	0.454	33	9% (3)	−1, 19	9% (3)	−1, 19	0.999	<0.001	<0.001
Any Anxiety Disorder	75	35% (26)	24, 46	27% (20)	16, 37	0.238	33	0% (0)	–	6% (2)	−3, 15	0.500	<0.001	0.018
Agoraphobia		8% (6)	2, 14	11% (8)	4, 18	0.727		0% (0)	–	0% (0)	–	–	0.174	0.103
Generalised Anxiety Disorder		8% (6)	2, 14	16% (12)	8, 24	0.070		0% (0)	–	0% (0)	–	–	0.174	0.016
OCD		3% (2)	−1, 6	0% (0)	–	0.500		0% (0)	–	0% (0)	–	–	0.999	–
Panic Disorder		3% (2)	−1, 6	3% (2)	−1, 6	0.999		0% (0)		7% (2)	1, 12	–	0.999	0.999
Separation Anxiety Disorder		5% (4)	0, 11	7% (5)	1, 12	0.999		0% (0)	–	0% (0)	–	–	0.311	0.320
Specific Phobia		17% (13)	9, 26	9% (7)	3, 16	0.070		0% (0)	–	3% (1)	−3, 9	0.999	0.009	0.430
Social Phobia		21% (16)	12, 31	16% (12)	8, 24	0.454		0% (0)	–	3% (1)	−3, 9	0.999	0.002	0.104
Any Mood Disorder	75	3% (2)	−1, 6	5% (4)	0, 11	0.625	33	0% (0)	–	3% (1)	−3, 9	0.999	0.999	0.999
Bipolar Disorder		0% (0)	–	0% (0)	–	–		0% (0)	–	0% (0)	–	–	–	–
Dysthymic Disorder		3% (2)	−1,6	4% (3)	−1, 9	0.999		0% (0)	–	3% (1)	−3, 9	0.999	0.999	0.999
Major Depressive Disorder		0% (0)	–	1% (1)	−1, 4	0.999		0% (0)	–	0% (0)	–	0.999	–	0.999
ADHD	75	37% (28)	26, 49	21% (16)	12, 31	**0.004**	33	3% (1)	−3, 9	3% (1)	−3, 9	0.999	<0.001	0.020
Conduct disorder	75	0% (0)	–	1% (1)	−1, 4	0.999	33	0% (0)	–	0% (0)	–	–	–	0.999
Oppositional Defiance Disorder	75	17% (13)	9, 26	15% (11)	6, 23	0.754	33	3% (1)	−3, 9	3% (1)	−3, 9	0.999	0.060	0.101
Any Psychotic disorder	75	0% (0)	–	0% (0)	–	–	33	0% (0)	−3, 9	0% (0)	–	–	–	–
Selective Mutism	75	4% (3)	−1, 9	4% (3)	−1, 9	0.999	33	3% (1)	−3, 9	3% (1)	−3, 9	0.999	0.999	0.551
Tic Disorder	75	5% (4)	0, 11	3% (2)	−1, 6	0.625	33	0% (0)	–	0% (0)	–	–	0.311	0.999
Trichtillomania	75	0% (0)	–	0% (0)	–	–	33	0% (0)	–	0% (0)	–	–	–	–
ASD screening (SCQ)	69	29% (22)	19, 40	38% (26)	26, 49	0.070	30	0% (0)	–	0% (0)	–	–	<0.001	<0.001

Prevalence of ADHD decreased from 37% (n = 28) at T1 to 21% (n = 16) at T2 in children with 22q11.2DS (McNemar's p = 0.004) (Table 1).

Level of medication use (5.3%, 4/75 children with 22q11.2DS) was low and had little effect on longitudinal change in diagnosis and the emergence of psychotic experiences (see Supplementary Materials), therefore medication was not taken into consideration in analyses.

### 5.2 Aim 2: factors associated with the emergence of psychotic experiences

Children with and without psychotic experiences as reported at T2 did not differ in terms of age, gender, 22q11.2DS inheritance, ethnicity, household income and maternal education and therefore these demographic variables were not included as covariates in subsequent analyses. All psychiatric and cognitive domains correlated positively with one another across T1 and T2 indicating that all scores had test-retest reliability (Supplementary Table 2), thus supporting the examination of change over time (Lowe and Rabbitt, 1998), as well as the analysis of level of impairment, in relation to the development of psychotic experiences.

### 5.3 Cognition

**Fig. 2:**
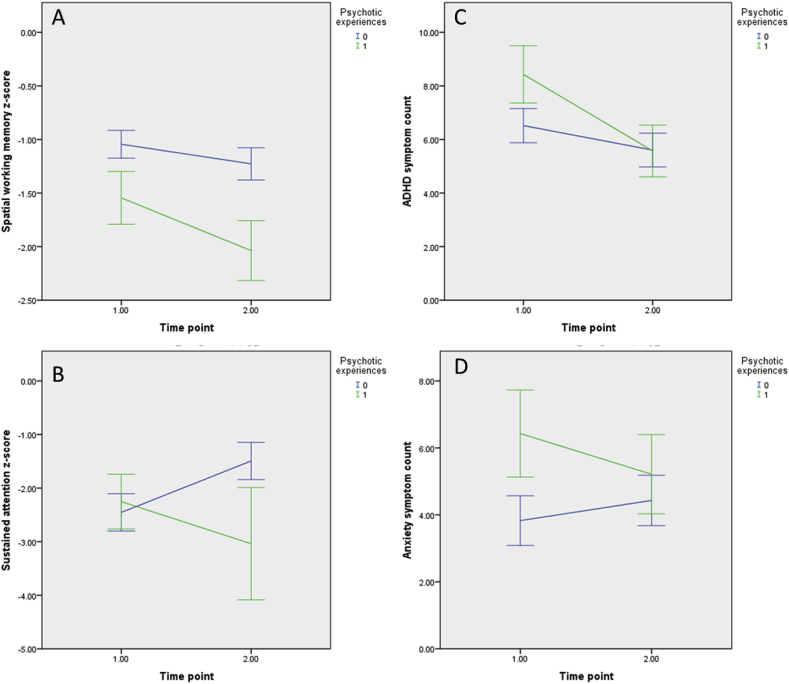
Relation of cognitive and psychopathology factors with psychotic experiences These graphs show the average trajectories of individuals with 22q11.2DS with and without psychotic experiences. Values for A and B can be found in Supplementary Table 3. Values for C and D can be found in Supplementary Table 4. Error bars represent the standard error.

Cognitive scores were only included if the child understood the task's instructions, therefore missingness was variable across neurocognitive measures (Supplementary Table 3). Mean performance in IQ combined for T1 and T2 (s_average_) and change in IQ (s_change_) were not associated with the emergence of psychotic experiences (Supplementary Table 3). Mean performance on spatial working memory combined for T1 and T2 (s_average_) was negatively associated (OR = 0.47, p = 0.034) with the emergence of psychotic experiences at T2. Individuals who had developed psychotic experiences by T2 (PE(+)) had a relative deficit of 0.50 SD in spatial working memory at T1 compared to the remainder of the sample (PE(-)) (Supplementary Table 3; Fig. 2, Panel A). Furthermore, deterioration in sustained attention was also associated with the emergence of psychotic experiences (OR = 5.25, p = 0.021); 64% (7/11) of the PE(+) group exhibited deterioration in sustained attention versus 25% (11/44) of the PE(-) group. The sustained attention of the PE(+) group was 0.20 SD above the PE(-) group at T1 but then dropped to 1.55 SD below the PE(-) group at T2 (Supplementary Table 3; see Fig. 2, Panel B).

### 5.4 Psychiatric features

Psychiatric diagnosis at T1 and T2 was not associated with emergence of psychotic experiences (Supplementary Table 4). When symptom counts were analysed, individuals who developed psychotic experiences at T2 had a greater total non-ASD symptom count (+6.41, p = 0.046) as well as anxiety symptom count (+2.60, p = 0.029) at T1, but this was no longer the case at T2 (Supplementary Table 4 and Fig. 2 Panel D). Additionally, ADHD symptom s_change_ was negatively associated with the emergence of psychotic experiences (OR = 0.54 (95%CI = 0.30–0.97, p = 0.039) (see Supplementary Table 4 and Fig. 2 Panel C). We included total symptom count on the CAPA (non-ASD symptomatology) in analyses, as previous studies on our cohort indicate a high level of psychiatric comorbidity (Cunningham et al., 2018; Niarchou et al., 2014) hence total count maybe better capture psychiatric severity, and also total symptom count on the CAPA has construct validity as total psychiatric symptom count as measured by the CAPA has previously been associated with increased probability of having used specialty mental health services (Angold and Costello, 2000).

A post-hoc analysis was conducted to investigate how much variance the factors identified accounted for. A logistic regression was conducted with spatial working memory s_average_, sustained attention deterioration, baseline anxiety symptoms and ADHD symptom s_change_ as predictors and emergence of psychotic experiences as the outcome. We did not include baseline non-ASD symptoms as this variable is mathematically related to baseline anxiety symptoms and ADHD symptom s_change_. Complete data for spatial working memory s_average_, sustained attention deterioration, baseline anxiety symptoms and ADHD symptom s_change_ was available for 55 children with 22q11.2DS, of which 11 had psychotic experiences. Nagelkerke r square was 0.353 (p = 0.006), indicating that the factors we identified accounted for 35.3% of the variance in psychotic experience outcome at T2. Only sustained attention deterioration (p = 0.037) and T1 anxiety symptoms (p = 0.049) were found to be unique contributors in the regression model. An important question is whether the factors identified represent processes that occur in the same individuals. To address this we tested if the factors identified were associated with each other. Spatial working memory ability s_average_ was found to be negatively correlated with the presence of sustained attention deterioration (r = −0.315, p- = 0.016), i.e. the lower an individual's spatial working memory ability, the more likely an individual was to exhibit deterioration in sustained attention. No other nominally significant correlations were found between the four predictors.

## 6 Discussion

This study provides a detailed examination of the longitudinal changes that occur in the crucial developmental period of early adolescence in 22q11.2DS. The same measures were administered at both time points and in cases and controls. Our study highlights that psychotic experiences in this high-risk syndrome emerge at an early age. Prevalence of subthreshold psychotic experiences increased in 22q11.2DS from 4% in childhood (T1) to 21% in early adolescence (T2), and at T2 prevalence was elevated relative to control siblings (3%). Hallucinatory phenomena particularly drove this increase. The majority of psychotic experiences in early adolescence were only reported by the child, highlighting that it is important for clinicians and researchers to conduct screening interviews for psychotic phenomena directly with children with 22q11.2DS in addition to interviewing the caregiver.

This initial emergence of psychotic experiences was associated with both neurocognitive and psychopathology factors. We did not find IQ ability or change in IQ to be related to the emergence of psychotic experiences, however, there was a link with the specific neurocognitive domains. Deficits in the spatial working memory aspect of executive function and deterioration in sustained attention were found to be associated with the emergence of psychotic experiences. Furthermore, these two processes were correlated with each other indicating that attention-executive function may be the initial neurocognitive domain altered in those who go on to develop psychotic disorder. Though it should be noted that not all our measures of executive function were associated with emergence of psychotic experiences, indicating that specific effects exist within broadly defined domains such as executive function.

There was high psychiatric morbidity in childhood and early adolescence in 22q11.2DS, prevalence of any psychiatric disorder, any non-ASD psychiatric disorder, any anxiety disorder, ADHD and putative ASD was elevated compared to control siblings. T1 non-ASD psychiatric symptomatology was found to be greater in children with 22q11.2DS who later developed psychotic experiences compared to children with 22q11.2DS who did not. Our measure of non-ASD psychiatric symptomatology has construct validity as total psychiatric symptom count as measured by the CAPA has previously been associated with increased probability of having used specialty mental health services (Angold and Costello, 2000). Furthermore, T1 level of anxiety symptoms was greater in children who later developed psychotic experiences.

The prevalence of ADHD decreased between T1 and T2, replicating previous findings for 22q11.2DS (Antshel et al., 2013) and idiopathic ADHD (Langley et al., 2010). Change in ADHD symptomatology was associated with the emergence of psychotic experiences at T2. This finding could be explained by diagnostic overshadowing, our measures of ADHD symptomatology and psychotic experiences may be indexing the same underlying trait that is interpreted differently with development.

Overall the neurocognitive and psychopathology factors we identified were found to account for 35.3% of the variance in psychotic experience outcome. Baseline anxiety symptoms and deterioration in sustained attention are particularly unique contributors. Future studies need to explore what factors explain the remaining variance.

### 6.1 Comparison to previous 22q11.2DS studies

Our study provides insights into the period of early adolescence in 22q11.2DS, and our sample size (n = 75) and time between assessments is comparable to previous longitudinal studies of 22q11.2DS (Antshel et al., 2010; Tang and Gur, 2017). Previous longitudinal studies have identified altered development in the attention-executive domain as contributing to the development of psychosis spectrum outcomes in 22q11.2DS (Hooper et al., 2013; Maeder et al., 2016; Tang and Gur, 2017). Our study highlights that these cognitive processes are occurring during early adolescence and are associated with the initial emergence of phenomena. Attention-executive deficits may be a domain that cuts across several neuropsychiatric features of 22q11.2DS, and may be more predictive of psychosis than individual neuropsychiatric features (Baker and Vorstman, 2012).

We do not find IQ to be related to emergence of psychotic experiences, however this may be because our study examines an earlier age period, when IQ trajectories are only hypothesised to begin to diverge in those who later develop psychosis. This may indicate that changes in global cognitive ability (IQ) may be secondary to initial changes in specific neurocognitive domains. Indeed other authors have hypothesised that executive function decline may contribute to VIQ decline seen in 22q11.2DS (Antshel et al., 2016). It brings into question whether IQ decline is a correlate of psychosis development rather than being truly antecedent. Discrepancy in findings could also reflect methodological differences. Our study used the WASI IQ test version, whereas previous studies have used the Wechsler Intelligence Scale for Children (WISC) which contains subtests that measure working memory and processing speed, therefore the WISC maybe more sensitive to neurocognitive domains that decline in 22q11.2DS than the WASI. Additionally, our study has a relatively narrow time gap which allows the detection of dynamic changes in cognitive and psychiatric development that may have otherwise been averaged out in studies with a wider time gap. However, a narrow time gap may not be as suitable to detect subtle changes that occur over a longer period.

Presence of anxiety at baseline has previously been identified as a risk factor for developing psychosis in 22q11.2DS (Gothelf, Feinstein, 2007a; Gothelf et al., 2013; Tang and Gur, 2017). This is in agreement with our finding that baseline anxiety symptom count is higher in those who later develop psychotic experiences. We did not find evidence that ASD (as screened by the SCQ) is related to emergence of psychotic phenomena, a finding consistent with previous 22q11.2DS studies that have found that ASD and psychosis are independent consequences of the deletion (Fiksinski et al., 2017; Vorstman et al., 2013).

### 6.2 Comparison to previous studies of psychotic phenomena in individuals without 22q11.2DS

Our findings are consistent with that observed in individuals with psychosis spectrum outcomes without 22q11.2DS. Several studies have implicated attention-executive deficits in the development of psychotic experiences and psychotic disorder in the general population (Niarchou et al., 2015; Reichenberg et al., 2010; Rossi et al., 2016). More generally, abnormalities of information processing and attention have played a central role in theories of psychosis since the time of Kraepelin and Bleuler (Braff, 1993). Also an increasing number of studies provide evidence suggesting that heightened anxiety is important in both the development of psychosis and maintaining psychosis (Hall, 2017). Overall this supports the conclusion that 22q11.2DS is a representative model for characterising childhood development associated with emergence of psychotic spectrum outcomes.

### 6.3 Limitations

Although we had the advantage of a longitudinal design and concurrent neurocognitive and psychiatric assessment at all time points, it is difficult to infer direction of effect with some of our findings. For instance, we are unable to unpick whether deterioration in sustained attention is a cause or correlate of the emergence of psychotic experiences. Not all children understood instructions for all cognitive tasks leading to missing data, and therefore findings may not be representative of children with 22q11.2DS and severe cognitive deficits. Our study is one of the few longitudinal studies that has examined neurocognitive ability beyond IQ, focusing on executive function, attention and processing speed. However we did not examine socio-emotional cognition, an important domain impaired in 22q11.2DS (Gur et al., 2014). We have argued that assessing the factors associated with the emergence of psychotic experiences may give insight into the initial phase of psychosis emergence, however it is important to emphasise that psychotic experiences, like other risk factors for psychotic disorder development, are not deterministic of developing full-blown psychotic disorder.

## 7 Conclusions

Early adolescence is the period of development in 22q11.2DS where early subthreshold psychotic experiences emerge. We identified neurocognitive and psychopathology factors associated with the emergence of psychotic experiences, and our findings suggest that the attention-executive domain and anxiety level are the first to be disrupted in the disease process that leads to psychotic disorder development in this high-risk group. Further work is needed to bring together the strands of 22q11.2DS research that have focused on different stages of psychosis development. Characterisation of these stages will give greater understanding on which factors contribute to disease progression and which are correlates of disease progression. 22q11.2DS is a powerful model to explore and understand the development of psychosis. Our findings may also have implications for psychosis risk in other high-risk populations.

## Conflicts of interest

No conflicts of interest to report.
