# Supplementary material for: The emergence of psychotic experiences in the early adolescence of 22q11.2 Deletion Syndrome
Source: J Psychiatr Res. 2019 Feb;109:10–7. doi: 10.1016/j.jpsychires.2018.11.002 (PMC6331974; doi:10.1016/j.jpsychires.2018.11.002)
Supplement: Supplementary Materials [file mmc1.docx]

**Supplementary Materials**

**Medication use**

Medication was reported by parents for 4 children with 22q11.2 Deletion Syndrome. Prescribed medication included; clonidine for ADHD, diazepam for anxiety, fluoxetine for anxiety, antipsychotic medication for aggressive outbursts. The child who was prescribed the fluoxetine, was the only child where prescribed medication resulted in them no longer meeting diagnostic criteria at T2. Psychotic experiences developed at T2 in one of these four children, the same child who was receiving fluoxetine for anxiety. Psychotic experiences were not present at T1 or T2 in the other three children.

**Supplementary Tables**

Supplementary Table 1: Psychotic experiences in children with 22q11.2DS and Controls

Values for columns 4 and 7 are McNemar p-values and indicate whether there was a significant change in prevalence between time points.

Supplementary Table 2: Cognition and psychopathology test-retest correlations of children with 22q11.2DS

FSIQ, Full Scale IQ; VIQ, Verbal IQ; PIQ, Performance IQ; ASD, Autism Spectrum Disorder; ODD, Oppositional Defiance Disorder.

Supplementary Table 3: Relations between cognition and psychotic experiences in children with 22q11.2DS

The PE(+) group consisted of individuals who had psychotic experiences at T2 but not at T1. The PE(-) group consisted of individuals who did not have psychotic experiences at T1 nor T2. Mean z-score (columns 3,4,7,8) is an age-adjusted standardised score, norms were not available for visual attention so z-scores could not be calculated. Individuals were classified as exhibiting deterioration if raw cognitive score decreased between time points (columns 5, 9). FSIQ, VIQ, PIQ do not have raw scores so for these measures individuals couldn’t be classified as showing raw score deterioration. Values in columns 10 and 11 are the mean z-score differences between PE(+) and PE(-) groups. The components s_average_ and s_change_ represent average cognitive ability and cognitive change respectively. Odds ratio (OR) and p-value metrics were derived using logistic regression, s_average_ and s_change_ were the predictor measures and PE status was the binary outcome measure (columns 12-15). A separate logistic regression was conducted with the presence of raw score deterioration as a binary predictor and PE status as the binary outcome measure (columns 16-17). See Statistical Analysis Aim 2 for full description of methods.

Supplementary Table 4: Relations between psychopathology and psychotic experiences in children with 22q11.2DS

The Any Psychiatric Disorder category was based on both the Child and Adolescent Psychiatric Assessment (CAPA) and the Social Communication Questionnaire (SCQ). Categorical diagnosis prevalence represents the frequency of individuals who met criteria for a psychiatric disorder on the CAPA or met the ASD screening cut-off on the SCQ. The Any Psychiatric Disorder symptom count is the total of the CAPA and SCQ symptom count. Any Non-ASD Psychiatric Disorder represents psychopathology reported on the CAPA and includes ADHD, anxiety, ODD and other symptoms covered by the CAPA. The PE(+) group consisted of individuals who had psychotic experiences at T2 but not at T1. The PE(-) group consisted of individuals who did not have psychotic experiences at T1 nor T2.Psychopathology was contrasted between PE(+) and PE(-) groups. R represents the rank correlation between diagnosis status (0,1) and psychotic experience status (0,1), providing a measure of effect size (columns 12 and 14). P-values in columns 16 and 17 indicate if there is a symptom count difference between PE(+) and PE(-) groups (Mann-Whitney U test). The components s_average_ and s_change_ represent average psychiatric symptom count and psychiatric symptom count change respectively. Odds ratio (OR) and p-value metrics were derived using logistic regression, where s_average_ and s_change_ were the predictor measures and PE status was the binary outcome measure (columns 18-21). See Statistical Analysis Aim 2 for full description of methods.
